# Enhancement of Cell Adhesion by *Anaplasma phagocytophilum* Nucleolin-Interacting Protein AFAP

**DOI:** 10.3390/jpm13020302

**Published:** 2023-02-08

**Authors:** Hongcheng Tang, Daxiu Zhang, Fenfen Jiang, Lifeng Yu, Hui Tang, Jiafeng Zhu, Shuyan Wu, Hua Niu

**Affiliations:** 1 Department of Microbiology, School of Biology & Basic Medical Sciences, Soochow University, Suzhou 215123, China; 2 Clinical Laboratory Center, Affiliated Hospital of Guilin Medical University, Guilin 541001, China; 3 Laboratory of Hepatobiliary and Pancreatic Surgery, Affiliated Hospital of Guilin Medical University, Guilin 541001, China; 4 Guangxi Key Laboratory of Molecular Medicine in Liver Injury and Repair, Guilin Medical University, Guilin 541001, China; 5 Guangxi Health Commission Key Laboratory of Basic Research in Sphingolipid Metabolism Related Diseases, Affiliated Hospital of Guilin Medical University, Guilin 541001, China

**Keywords:** *Anaplasma phagocytophilum*, type IV secretion system, cell adhesion, nucleolin, actin filaments, podosomes

## Abstract

*Anaplasma phagocytophilum*, the aetiologic agent of human granulocytic anaplasmosis (HGA), is an obligate intracellular Gram-negative bacterium. During infection, *A. phagocytophilum* enhances the adhesion of neutrophils to the infected endothelial cells. However, the bacterial factors contributing to this phenomenon remain unknown. In this study, we characterized a type IV secretion system substrate of *A. phagocytophilum*, AFAP (an actin filament-associated *Anaplasma phagocytophilum* protein) and found that it dynamically changed its pattern and subcellular location in cells and enhanced cell adhesion. Tandem affinity purification combined with mass spectrometry identified host nucleolin as an AFAP-interacting protein. Further study showed the disruption of nucleolin by RNA interference, and the treatment of a nucleolin-binding DNA aptamer AS1411 attenuated AFAP-mediated cell adhesion, indicating that AFAP enhanced cell adhesion in a nucleolin-dependent manner. The characterization of cell adhesion-enhancing AFAP and the identification of host nucleolin as its interaction partner may help understand the mechanism underlying *A. phagocytophilum*-promoting cell adhesion, facilitating the elucidation of HGA pathogenesis.

## 1. Introduction

*Anaplasma phagocytophilum*, a Gram-negative obligatory intracellular bacterium (rickettsia), causes the emerging tick-borne zoonosis called human granulocytic anaplasmosis (HGA), which is characterized by fever, malaise, headache, myalgia, arthralgia, leukopenia, thrombocytopenia, and elevations in serum hepatic aminotransferases [1]. *A. phagocytophilum* infects midgut cells, hemocytes, and salivary gland cells in ticks and endothelial cells and neutrophils in mammalians [2,3,4]. *A. phagocytophilum* infection causes multiple physiological changes in host cells, including actin cytoskeleton remodeling and altered cell adhesion [2,4,5,6,7,8,9]. It was proposed that after the tick bite, endothelial cells serve as reservoirs for *A. phagocytophilum* and pass it on to neutrophils for dissemination and persistence in mammalian hosts [4,9]. The efficient transmission of *A. phagocytophilum* from endothelial cells to neutrophils may rely on cell interaction. It was showed that during infection, *A. phagocytophilum* enhances the adhesion of neutrophils to infected endothelial cells [4,9]. However, bacterial factors contributing to the enhanced cell adhesion remain unknown.

Cell adhesion is mediated by adhesion molecules located on the cell surface, which generally belong to five families: cadherins, integrins, selectins, immunoglobulin superfamily, and others [10]. These adhesion molecules engage their partners on other cells or extracellular matrices to form junctional contact or nonjunctional contact, promoting cells to move, communicate, differentiate, or assemble to form tissues and organs. Meanwhile, with the linking to adhesion molecules via anchor proteins, cytoskeleton strengthens cell adhesion [11]. There are several types of cell junctions, such as tight junctions, adherens junctions, desmosomes, and gap junctions in cell–cell adhesion and hemidesmosomes, focal adhesion, and podosomes in cell–matrix adhesion [12,13,14,15,16]. These cell junctions play an important role in innate defense, as exemplified by the impermeability of epithelia, which blocks pathogen penetration, and the exfoliation of mucosal epithelial cells, which inhibits bacterial colonization [17,18]. However, pathogens can manipulate cell adhesion to benefit their infection [19,20]. For instance, *Shigella flexneri* translocates its effector OspE into epithelial cells to enforce focal adhesion by interacting with host integrin-linked kinase, thereby inhibiting cell detachment, and promoting bacterial colonization [21]; *Listeria* secretes protein InlC into the cytosol of infected epithelial cells, interacting with host protein Tuba to remodel cell–cell junctions, facilitating its spreading among host cells [22]; and HIV uses podosomes to invade macrophages [23].

AFAP is a type IV secretion system effector of *A. phagocytophilum* and was found to be associated with host actin filaments, thereby named as an actin filament-associated *Anaplasma phagocytophilum* protein [24]. Here, we found AFAP localized to cell periphery, surrounded punctate F-actin-rich cores and enhanced cell adhesion. Tandem affinity purification combined with mass spectrometry identified host nucleolin as an interaction partner of AFAP. Further study showed that the disruption of nucleolin attenuated AFAP-mediated cell adhesion, indicating that AFAP enhanced cell adhesion, likely through interaction with nucleolin. This study may help understand the mechanism underlying *A. phagocytophilum*-promoting cell adhesion, facilitating the elucidation of HGA pathogenesis.

## 2. Materials and Methods

### 2.1. Cell Cultures and Plasmids

HeLa cells, HEK293 cells, and EA.hy926 cells were propagated in Dulbecco’s modified Eagle’s medium (DMEM) (Gibco of Thermo Fisher Scientific, Waltham, MA, USA) supplemented with 10% fetal bovine (Gibco) at 37 °C and 5% CO_2_. Plasmids pAFAP-GFP and pLifeact-mCherry were constructed in a previous study [24]. Plasmid pEGFP-N1, which expresses GFP, was from Clontech Laboratories. Plasmid pTAP-AFAP was constructed by cloning the gene fragment encoding the AFAP–streptavidin-binding peptide-3×FLAG tag fusion protein (AFAP-SBP-3×FLAG, shorted as AFAP-SF) into the mammalian expression vector pIRESpuro3 (Takara, San Jose, CA, USA). SBP is a peptide with the length of 38 amino acids [25]; 3×FLAG is an epitope tag with the length of 22 amino acids [26]. The DNA fragment encoding SBP-3×FLAG was chemically synthesized and ligated to an AFAP-coding DNA fragment, generated by PCR using pAFAP-GFP as a template, to obtain the AFAP-SF-coding DNA fragment. Plasmid pTAP, which only expresses the streptavidin-binding peptide-3×FLAG tag fusion protein (SBP-3×FLAG, shorted as SF), was constructed by the deletion of the AFAP-coding sequence from pTAP-AFAP. The construction of the plasmids pTAP-AFAP and pTAP was completed by GENEWIZ, Inc. (Suzhou, China). Plasmid pmCherry-nucleolin was constructed by cloning the DNA fragment encoding nucleolin into the mammalian expression vector pmCherry-C1 (Takara). The DNA fragment encoding nucleolin was commercially synthesized (Genscript, Nanjing, China), and the recombination-based cloning was performed with the ClonExpress Ultra One Step Cloning Kit (Vazyme, Nanjing, China) according to the manufacturer’s instructions. Briefly, linearized pmCherry-C1 was obtained by PCR using a pair of primers (pmCherry-F: 5′-GGATCCACCGGATCTAGATAACTG-3′ and pmCherry-R: 5′-GTCGACTGCAGAATTCGAAGC-3′), with the pmCherry-C1 plasmid as the template; a DNA fragment encoding nucleolin was extended by PCR using a pair of primers (NCL-F: 5′- CTTCGAATTCTGCAGTCGACATGGTGAAGCTCGCGAAGG-3′ and NCL-R: 5′-TATCTAGATCCGGTGGATCCTTATTCAAACTTCGTCTTCTTTCCTT-3′) to add sequences, which matched with the termini of linearized pmCherry-C1. PCR products were purified and mixed together with 2 × ClonExpress Mix, which contained the recombinase Exnase. The recombination reaction was performed at 50 °C for 5 min, and the recombinant product was used for transformation into the *Escherichia coli* DH5α strain to generate pmCherry-nucleolin.

### 2.2. Transfection, Tandem Affinity Purification, and Coimmunoprecipitation

To determine the subcellular localization of AFAP and actin filaments, plasmid pAFAP-GFP or pEGFP-N1 was transfected into HeLa cells, together with pLifeact-mCherry. To determine the colocalization of AFAP-GFP with mCherry-nucleolin, EA.hy926 cells were transfected with plasmids pAFAP-GFP and pmCherry-nucleolin. To perform tandem affinity purification, HEK293 cells were transfected with plasmid pTAP-AFAP or pTAP, respectively. Briefly, cells at 70% confluent in a well of 6-well plates were transfected by using the Lipofectamine 3000 transfection reagent (Invitrogen, Waltham, MA, USA) (3.75 μL of Lipofectamine 3000 reagent, 5 μL of P3000 reagent, and 2.5 μg of DNA (1.25 μg DNA for each plasmid in cotransfection)) according to manufacturer’s instruction. Transfected cells were harvested or further treated at a designated time. To generate stable transfectants expressing AFAP-SF or SF, transfected HEK293 cells were cultured in complete DMEM medium containing 0.5 μg/mL puromycin (Thermo Fisher Scientific, Waltham, MA, USA) from the 5th day post-transfection. To perform tandem affinity purification, HEK293 cells stably transfected with pTAP-AFAP or nontransfected HEK293 cells were propagated in eight 150 mm^2^ cell culture dishes (~4 × 10^8^ cells) and lysed in lysis buffer (30 mM Tris-HCl, 150 mM NaCl, and 0.5% (*v*/*v*) nonidet-P40, pH 7.4) (1.2 mL/dish) supplemented with a protease inhibitor cocktail and phosphatase inhibitors (APExBIO, Houston, TX, USA). The cell lysates were cleared by centrifugation at 12,000× *g* for 10 min at 4 °C, and the supernatants were subjected to incubation with streptavidin resin (GenScript, Nanjing, Jiangsu, China) (50 μL/dish) for 2 h at 4 °C, followed by washing 4 times with the washing buffer (30 mM Tris-HCl, 150 mM NaCl, and 0.1% (*v*/*v*) nonidet-P40, pH 7.4), which was supplemented with the protease inhibitor cocktail and phosphatase inhibitors, and elution with 1.2 mL of elution buffer (30 mM Tris-HCl, 150 mM NaCl, and 4 mM biotin, pH 7.4). The eluates were further purified with magnetic beads conjugated with mouse anti-FLAG antibody (Bimake, Shanghai, China) (15 μL/dish). After incubation for 2 h at 4 °C, the beads were washed with washing buffer once and TBS (30 mM Tris-HCl, 150 mM NaCl, pH 7.4) twice, followed by elution with 60 μL of non-reducing 2 x SDS-PAGE sample loading buffer (100 mM Tris-Cl, 20% (*v*/*v*) glycerol, 4% (*w*/*v*) SDS, and 0.2% (*w*/*v*) bromophenol blue, pH 6.8). The eluates were subjected to SDS-PAGE, and protein bands of interest were commercially submitted for mass spectrometry to identify the protein (BiotechPack Scientific, Beijing, China). Briefly, 20 μL of eluates were subjected to SDS-PAGE analysis, followed by staining with Coomassie blue G-250 solution. The gel slices trimmed to contain the major band of interest were dehydrated with acetonitrile and reduced with 10 mM DTT, followed by in-gel digestion with trypsin (Promega, Madison, WI, USA). The extracted peptide fragments were desalted and subjected to nano LC-MS/MS analysis, which was performed in an EASY-nLC1200 nanoflow HPLC system (ThermoFisher Scientific) and a Q Exactive Hybrid Quadrupole-Orbitrap Mass Spectrometer (ThermoFisher Scientific). The raw MS data were analyzed and searched against the human protein database using the MaxQuant mass spectrometry analysis software. The identified protein with the biggest spectral count in mass spectrometry was assigned to the major band in the SDS-PAGE gel slice.

For coimmunoprecipitation, HEK293 cells stably transfected with pTAP-AFAP from two wells of a 6-well plate (~6 × 10^6^ cells) were lysed in 1 mL of immunoprecipitation buffer (IP) (50 mM HEPES, 150 mM KCl, 1 mM EDTA, 1.0% (*v*/*v*) Triton X-100, 10% (*v*/*v*) glycerol, pH 7.4) supplemented with a protease inhibitor cocktail (APExBIO). After cell lysate was cleared by centrifugation at 16,000× *g* for 10 min at 4 °C, the supernatant was subjected to coimmunoprecipitation in a tube containing 2 μg of rabbit polyclonal anti-FLAG antibody (Bioworld Technology, Bloomington, MN, USA), rabbit polyclonal anti-HA tag antibody (BBI Life Sciences, Shanghai, China), mouse monoclonal anti-nucleolin antibody (D-6, Santa Cruz Biotechnology (SCBT), Dallas, TX, USA), or mouse normal IgG (SCBT). After incubation at 4 °C for 2 h, 20 μL of protein A agarose (SCBT) was added into each tube. Protein A agarose resin was washed 4 times with IP buffer and eluted by boiling for 5 min in 40 μL of 2 × SDS-PAGE sample loading buffer containing 200 mM DTT. An amount of 10 μL of the precipitates were probed in a Western blot analysis using mouse monoclonal anti-nucleolin antibody (D-6) and rabbit polyclonal anti-FLAG antibody.

### 2.3. Immunofluorescence Labeling and Confocal Microscopy

For the determination of cell surface localization of nucleolin in AFAP-GFP-expressing HeLa cells, HeLa cells transfected with pAFAP-GFP for 3 d were subjected to incubation at RT for 1 h with mouse monoclonal anti-nucleolin antibody (MS-3, SCBT) diluted in DMEM medium at 1:20. After washing with DMEM medium 3 times, cells were fixed with 4% paraformaldehyde at RT for 20 min. Fixed cells were washed 3 times with 1 × PBS (137 mM NaCl, 2.7 mM KCl, 10 mM Na_2_HPO_4_, and 2 mM KH_2_PO_4_, pH 7.4) and blocked in 1 × PBS containing 0.8% BSA for 10 min, followed by incubation at 37 °C for 45 min with Alexa Fluor 555-conjugated goat anti-mouse antibody (Invitrogen). These immunolabeled cells were observed under a Leica TCS SP8 confocal microscope (Leica, Wetzlar, Germany). Other cells transfected with fluorescent protein plasmids were fixed with 4% paraformaldehyde at RT for 20 min and subjected to observation under a Leica TCS SP8 confocal microscope (Leica) or a Nikon Eclipse Ti2 confocal microscope.

### 2.4. RNA Interference and Treatment with AS1411 Aptamer

For RNA interference, HEK293 cells stably transfected with pTAP-AFAP were transfected with siRNA against nucleolin mRNA and control siRNA A (SCBT) using the Lipofectamine RNAiMAX transfection reagent (Invitrogen) according to the manufacturer’s instructions. Briefly, the day before transfection, 1 × 10^4^ cells were seeded into a well of a 96-well plate. An amount of 1 pmol of each siRNA was added into the HEK293 cells after the formation of the siRNA–lipid complex. The cells were further incubated for 3 d, followed by cell detachment assay and Western blot analysis using mouse monoclonal anti-nucleolin antibody (D-6, SCBT) and mouse monoclonal anti-β-actin antibody (Beyotime Biotechnology, Shanghai, China).

AS1411 aptamer, which is a guanosine-rich oligonucleotide, specifically targets cell surface nucleolin [27]. AS1411 (5′-GGTGGTGGTGGTTGTGGTGGTGGTGG-3′) and its negative control CRO (cytosine-rich oligonucleotide) (5′-CCTCCTCCTCCTTCTCCTCCTCCTCC-3′) were chemically synthesized, desalted (GENEWIZ), reconstituted in pure water at a concentration of 500 μM, and filtered through a 0.22 μm filter. AS1411 or CRO was added into the 2 × 10^4^ HEK293 cells in a well of a 96-well plate at a final concentration of 2, 6, and 10 μM, respectively. After incubation for 4 d, the cells were subjected to cell detachment assay.

### 2.5. Cell Detachment Assay

Transfected HEK293 cells in 96-well plates were washed with prewarmed 1 × PBS after cultivation for 4 to 6 d, followed by incubation with 2 μM calcein-AM (Beyotime Biotechnology) in DMEM medium for 30 min. After the DMEM medium containing calcein-AM was replaced with fresh DMEM medium, HEK293 cells were incubated for another 30 min. After washing twice with prewarmed 1 × PBS, the plates were sealed with plate covers and centrifuged upside down at 100× *g* for 5 min at 4 °C. Floating cells were carefully removed, and the wells were refilled with 1 × PBS. Cells were imaged, and the fluorescence intensity in the wells was measured in a plate reader (BioTek, Winooski, VT, USA) with an excitation wavelength of 494 nm and an emission wavelength of 514 nm before and after centrifugation.

### 2.6. Western Blot Analysis

HEK293 cells or immunoprecipitates in reducing 2 × SDS-PAGE sample loading buffer were subjected to SDS-PAGE, with 10% polyacrylamide resolving gels. Proteins were transferred to nitrocellulose membranes using a Mini Trans-Blot cell (Bio-Rad, Hercules, CA, USA) according to the manufacturer’s instructions, and the membranes were probed with primary antibodies, including mouse monoclonal anti-nucleolin (1:1000 dilution), rabbit anti-FLAG (1:1000 dilution), or mouse monoclonal anti-β-actin (1:1000 dilution), at RT for 1 h. β-actin was used as an internal control for sample loading in RNA interference. After washing three times with 1 × PBS (10 min each time), the membranes were incubated with secondary antibody, peroxidase-conjugated goat anti-mouse IgG (1:2000 dilution), or peroxidase-conjugated goat anti-rabbit IgG (1:2000 dilution) (KPL, Gaithersburg, MD, USA) at RT for 1 h. The membranes were washed four times with 1 × PBS (10 min each time) and subjected to ECL chemiluminescence. The membranes were imaged by the Tanon 4200 chemiluminescence imaging system (Tanon, Shanghai, China). The band intensities were determined with image processing software ImageJ (NIH, Bethesda, MD, USA).

### 2.7. Statistical Analysis

More than three independent experiments were conducted, and data were expressed as mean  ±  SEM. Statistical significance was evaluated by Student’s *t*-test.

### 2.8. GenBank Accession Numbers

pTAP-AFAP: OP292671; pTAP: OP292672.

## 3. Results

### 3.1. AFAP Dynamically Changed Its Pattern and Subcellular Location in Transfected Cells

**Figure 1:**
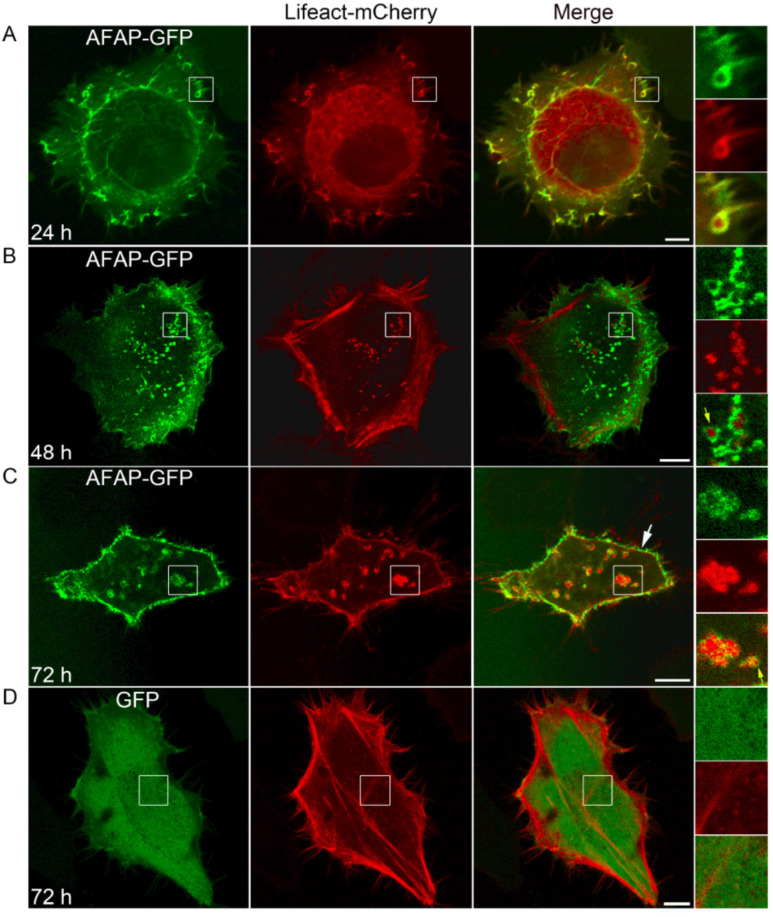
The dynamical changes of pattern and subcellular location of AFAP. HeLa cells were cotransfected with plasmids expressing AFAP-GFP and Lifeact-mCherry, followed by observation under a confocal fluorescence microscope at 24 h post-transfection (**A**), 48 h post-transfection (**B**), and 72 h post-transfection (**C**). Alternatively, HeLa cells were cotransfected with plasmids expressing GFP and Lifeact-mCherry, followed by observation at 72 h post-transfection (**D**). Lifeact: an F-actin-binding peptide. The boxed areas are magnified on the right. Yellow arrows indicate the structures with two-part architecture, in which F-actin cores were surrounded by AFAP rings. White arrow indicates cell periphery. Scale bars: 10 μm.

As previously reported [24], AFAP was found colocalized with the actin filaments at 24 h post-transfection in HeLa cells (Figure 1A). However, with the extended incubation time, a population of AFAP formed ring-like structures, which encircled punctate F-actin-rich cores, indicated by the F-actin-binding Lifeact at 48 and 72 h post-transfection (Figure 1B,C). The structures presented as F-actin-rich cores surrounded by AFAP rings were morphologically similar to those of podosomes. Podosomes are adhesion structures at the ventral surface of cells from the myeloid lineage and the endothelial lineage with two-part architecture, in which F-actin-rich cores are surrounded by rings composed of adhesion plaque proteins and integrins [16,28]. Moreover, it was also observed that another population of AFAP localized to the cell periphery with actin filaments at 48 and 72 h post-transfection (Figure 1B,C). This result indicates that AFAP dynamically changed its pattern and subcellular location in transfected cells over time. Furthermore, the stress fibers were clearly observed in GFP-expressing cells (Figure 1D) but rarely seen in AFAP-GFP-expressing cells at 72 h post-transfection (Figure 1B,C), suggesting that AFAP may cause the remodeling of the actin cytoskeleton.

### 3.2. Nucleolin Was Identified as an AFAP-Interacting Protein

**Figure 2:**
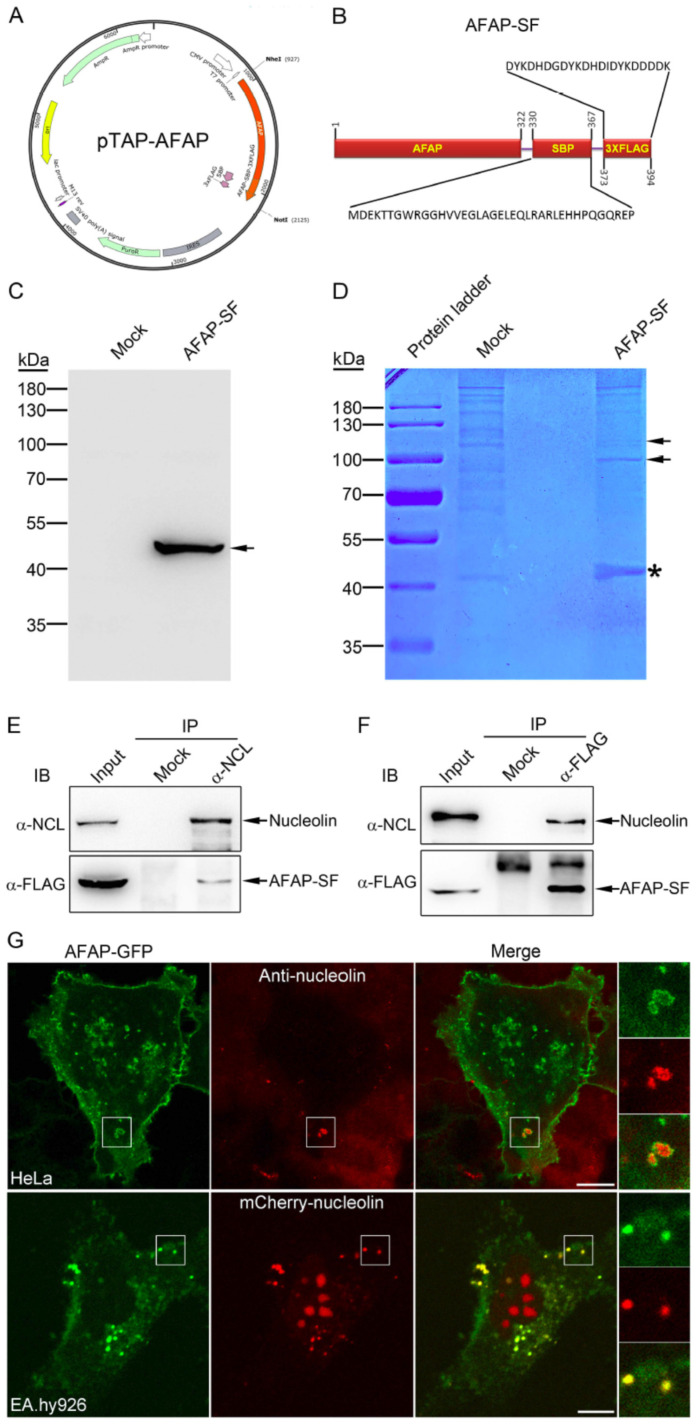
Identification of host nucleolin as an AFAP-interacting protein. (**A**) Construction of plasmid pTAP-AFAP expressing AFAP-SBP-3×FLAG (AFAP-SF). The gene encoding AFAP-SBP-3×FLAG was chemically synthesized, followed by cloning into plasmid pIRESpuro3 to generate pTAP-AFAP. (**B**) Diagram of the AFAP-SBP-3×FLAG fusion protein (AFAP-SF). SBP (streptavidin-binding peptide): a peptide that is 38 amino acids in length; 3×FLAG, a peptide tag that is 22 amino acids in length. The numbers indicate the start position and end position for each designated protein or peptide. (**C**) Western blot analysis for AFAP-SF in HEK293 cells transiently transfected with pTAP-AFAP (AFAP-SF). Mock: nontransfected HEK293 cells. Arrow: AFAP-SF band. (**D**) Tandem affinity purification of AFAP-interacting proteins and mass spectrometry. HEK293 cells stably transfected with pTAP-AFAP (AFAP-SF) and HEK293 cells (mock) subjected to tandem affinity purification with streptavidin resin and magnetic beads conjugated with mouse anti-FLAG antibody after lysis. Eluted proteins were resolved by SDS-PAGE. Asterisk: AFAP-SF band; arrows: bands present in the AFAP-SF lane but absent in the mock lane. The bands indicated with arrows were subjected to mass spectrometry, and the upper band was identified as nucleolin. (**E**,**F**) Coimmunoprecipitation assay for the interaction between AFAP and nucleolin. The cell lysates (Input) from HEK293 cells stably transfected with pTAP-AFAP (AFAP-SF) were subjected to immunoprecipitated (IP) with mouse normal IgG (mock) (**E**), mouse monoclonal anti-nucleolin (α-NCL) (**E**), rabbit polyclonal anti-HA antibody (mock) (**F**), or rabbit polyclonal anti-FLAG antibody (α-FLAG) (**F**). Immunoprecipitates were immunoblotted (IB) with mouse monoclonal anti-nucleolin (α-NCL) and rabbit polyclonal anti-FLAG antibody (α-FLAG). (**G**) Colocalization assay for AFAP and nucleolin. HeLa cells were transfected for 3 d with plasmids expressing AFAP-GFP, followed by incubation with mouse monoclonal anti-nucleolin antibody and Alexa Fluor 555-conjugated goat anti-mouse IgG without permeabilization of the cell membrane; EA.hy926 cells were cotransfected with plasmids expressing AFAP-GFP and plasmids expressing mCherry-nucleolin for 2 d. After fixation, cells were subjected to confocal microscopy. The boxed areas of interest were magnified on the right. Scale bars: 10 μm.

To identify host proteins interacting with AFAP, tandem affinity purification combined with mass spectrometry was employed. Plasmid pTAP-AFAP, which expresses the AFAP–streptavidin-binding peptide-3×FLAG tag fusion protein (AFAP-SF), was constructed (Figure 2A). AFAP-SF is a protein with a length of 394 amino acids, including the 38-amino-acid streptavidin-binding peptide and the 22-amino-acid 3×FLAG tag (Figure 2B). When AFAP-SF was expressed in transfected cells, it showed an expected size of 41.1 kDa (Figure 2C). AFAP-SF-interacting proteins were isolated by tandem affinity purification and resolved by SDS-PAGE. Besides the AFAP-SF band, there were two extra bands with sizes corresponding to 110 and 100 kDa, respectively, in the eluate from HEK293 cells stably expressing AFAP-SF, compared to the eluate from nontransfected HEK293 cells (Figure 2D). Although the band with the size of 100 kDa remained unidentified, the band with the size of 110 kDa was assigned as nucleolin by mass spectrometry based on spectral counting. The following Western blot analysis using monoclonal antibody against nucleolin showed the presence of nucleolin in the AFAP-SF lane and the absence of nucleolin in the mock lane, validating the result of the mass spectrometry (data not shown). To confirm the interaction between AFAP and nucleolin, coimmunoprecipitation was performed. Both anti-FLAG antibody and anti-nucleolin antibody precipitated the AFAP-SF and nucleolin (Figure 2E,F), indicating that there was interaction between AFAP and nucleolin. To exclude the possibility that the interaction between AFAP-SF and nucleolin in tandem affinity purification and co-immunoprecipitation was through the SF tags in AFAP-SF, we performed a pull-down assay from cell lysates of the AFAP-SF-expressing HEK293 cells and two negative controls (SF tag-expressing HEK293 cells and APH0215-SF-expressing HEK293 cells) using anti-FLAG tag magnetic beads. We found that nucleolin was pulled down only from AFAP-SF-expressing cells but not from SF tag-expressing cells and APH0215-SF-expressing cells, indicating that the interaction between AFAP and nucleolin was specific (Figure S1). Of note, APH0215 is an *A. phagocytophilum* protein [29]. APH0215-SF, which harbors SBP-3×FLAG, was constructed (Yu et al., unpublished data) and used here as a negative control.

Nucleolin is a multifunctional protein participating in a variety of cell functions such as ribosome biogenesis, transcriptional regulation, cell proliferation, and apoptosis [30]. While nucleolin is mainly found in the nucleus and cytoplasm, it has also been found on the cell surface [31,32]. To determine whether the cell surface is one location at which AFAP interacted with nucleolin, immunofluorescence labeling for cell surface nucleolin was performed in AFAP-GFP-expressing HeLa cells. Cell surface nucleolin was found colocalized with AFAP-GFP (Figure 2G), indicating that cell surface was a subcellular location for the interaction between AFAP and nucleolin. To further strengthen the observation that AFAP-GFP colocalized with nucleolin, the colocalization of AFAP-GFP with mCherry-nucleolin was determined in the immortalized human umbilical vein endothelial cells, EA.hy926 [33]. Due to their flat shape, endothelial cells are suitable for colocalization assay. Meanwhile, endothelial cells are permissive for *A. phagocytophilum* infection [34]. EA.hy926 cells were cotransfected with plasmids pAFAP-GFP and pmCherry-nucleolin for 2 d and subjected to confocal microscopy. mCherry-nucleolin was found localized to nucleoli and extranuclear regions (Figure 2G). Furthermore, extranuclear mCherry-nucleolin was found colocalized with AFAP-GFP (Figure 2G). These results indicate that nucleolin is a host AFAP-interacting protein.

### 3.3. AFAP Enhanced Cell Adhesion in a Nucleolin-Dependent Manner

**Figure 3:**
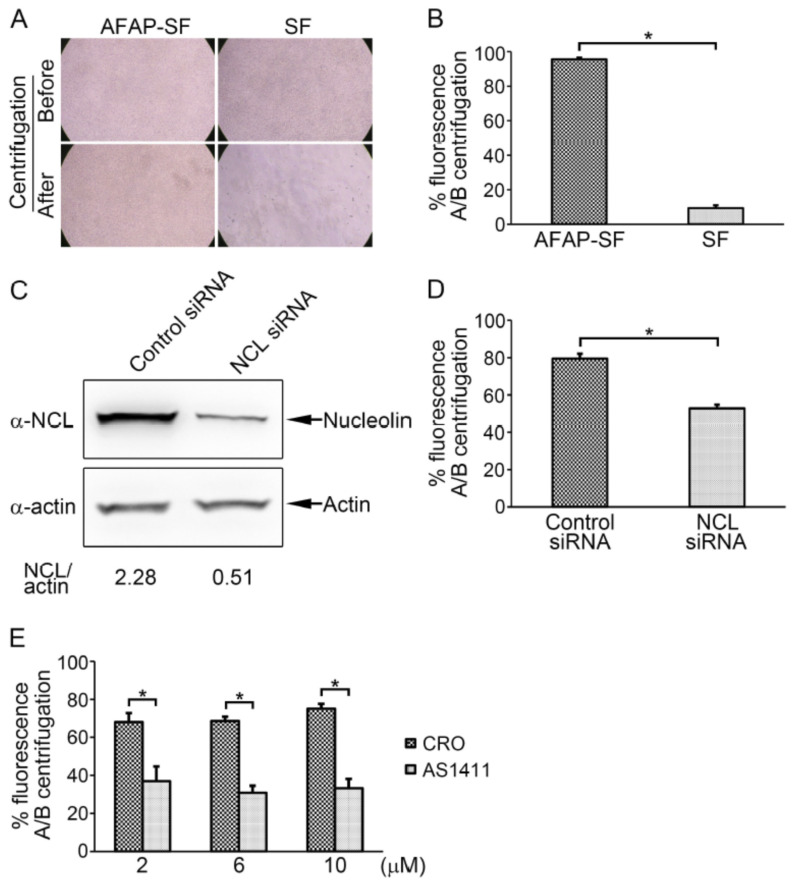
AFAP enhanced cell adhesion in a nucleolin-dependent manner. (**A**) The cell images of HEK293 cells stably transfected with pTAP-AFAP (AFAP-SF) or pTAP (SF) were captured under a light microscope (×100 magnification) before and after centrifugation. (**B**) Percentages of fluorescence intensity (fluorescence) of calcein in wells after centrifugation/before centrifugation (A/B centrifugation) in cell detachment assay. AFAP-SF: HEK293 cells stably transfected with pTAP-AFAP. SF: HEK293 cells stably transfected with pTAP. *: Significant difference (*p* < 0.01) between groups indicated with lines by Student’s *t*-test. (**C**) Treatment with nucleolin siRNA reduced nucleolin expression. HEK293 cells stably expressing AFAP-SF were transfected with control siRNA or nucleolin siRNA (NCL siRNA) for 3 d, followed by Western blot analysis using mouse anti-nucleolin (α-NCL) and anti-β-actin (α-actin) antibodies. Actin was used as an internal control to normalize the sample loading amount. Relative intensity ratios of nucleolin (NCL)/actin bands are shown below each lane. (**D**) Treatment with nucleolin siRNA attenuated AFAP-mediated cell adhesion. HEK293 cells stably expressing AFAP-SF were transfected with control siRNA or nucleolin siRNA (NCL siRNA) for 3 d, followed by cell detachment assay. Percentages of fluorescence intensity (fluorescence) of calcein in wells after centrifugation/before centrifugation (A/B centrifugation) were calculated. *: Significant difference (*p* < 0.01) between groups indicated with lines by Student’s *t*-test. (**E**) Treatment with cell surface nucleolin-targeting aptamer AS1411 attenuated AFAP-mediated cell adhesion. HEK293 cells stably expressing AFAP-SF were treated with control DNA oligonucleotide (CRO) or aptamer AS1411 at designated concentrations for 4 d, followed by cell detachment assay. Percentages of fluorescence intensity (fluorescence) of calcein in wells after centrifugation/before centrifugation (A/B centrifugation) were calculated. *: Significant difference (*p* < 0.05) between groups indicated with lines by Student’s *t*-test.

Cell surface nucleolin binds a multitude of ligands, such as laminin-1 and L-selectin, mediating cell–matrix adhesion and cell–cell adhesion [35,36]. Meanwhile, AFAP interacts with nucleolin. Thus, the effect of AFAP on cell adhesion was investigated. HEK293 cells have been employed for cell adhesion and migration studies [37,38]. While HEK293 cells are loosely adherent, which means they are easy to dissociate from plastic surfaces by force, HEK293 cells stably expressing AFAP-SF were difficult to dissociate from the culture flask by pipetting in the routine cell passaging, especially with an extended incubation time. Trypsin treatment had to be employed to completely dissociate the cells from the culture flask when the cells were cultured for more than 6 d. To determine the effect of AFAP on cell adhesion in a quantitative way, cell detachment assay was employed, which uses the centrifugation force to detach cells from plastic surfaces [39,40]. No obvious cell dissociation was observed under a light microscope after centrifugation in wells, in which HEK293 cells stably transfected with pTAP-AFAP (AFAP-SF) were cultured for 6 d, compared to HEK293 control cells stably transfected with pTAP (SF), which only had scattered cells in wells after centrifugation (Figure 3A and Figure S2). Calcein-AM is a cell-permeant dye that is converted to green-fluorescent calcein by hydrolysis with intracellular esterases. Due to the polar nature of calcein, it is retained within the cells. Thus, the fluorescence intensity can be used as an indicator for cell number in cell adhesion assays [41]. It was found that 95.6 ± 1.0% fluorescence intensity of calcein remained in these AFAP-SF wells, compared to 9.3 ± 1.7% fluorescence intensity in SF wells, after centrifugation (Figure 3B). This result indicates that AFAP enhanced cell adhesion.

Since AFAP interacted with nucleolin, the effect of nucleolin on AFAP-mediated cell adhesion was determined. The siRNA-mediated knockdown of nucleolin was performed. Compared to the siRNA negative control, siRNA treatment for nucleolin reduced its expression and cell adhesion, as shown by Western blot analysis and cell detachment assay (Figure 3C,D). Quantification of the band intensities of Western blots showed that RNA interference with siRNA reduced nucleolin expression by 78% (Figure 3C). Meanwhile it was found that 79.5 ± 2.7% fluorescence intensity of calcein remained in wells of culture plates treated with siRNA negative control versus 52.8 ± 2.0% fluorescence intensity of calcein in the wells of culture plates treated with nucleolin siRNA after centrifugation in cell detachment assay (Figure 3D). To corroborate this result, treatment with a DNA aptamer, AS1411, was performed. AS1411, a G-rich oligonucleotide, targets cell surface nucleolin and exerts its effect, such as the inhibition of cell proliferation, blocking the binding of nucleolin to its ligands [27,42]. HEK293 cells stably expressing AFAP-SF were treated with AS1411 or its negative control, CRO, at 2, 6, and 10 μM for 4 d. Compared to CRO, cells treated with AS1411 at 2, 6, and 10 μM were less adherent to wells after centrifugation in cell detachment assay (67.1 ± 4.8% vs. 35.8 ± 7.6% at 2 μM; 67.8 ± 2.3% vs. 29.6 ± 3.7% at 6 μM; 74.2 ± 2.6% vs. 32.1 ± 4.9% at 10 μM) (Figure 3E), indicating that AS1411 attenuated AFAP-mediated cell adhesion. Taken together, these results showed that the disruption of nucleolin attenuated the enhancement effect of AFAP on cell adhesion, suggesting AFAP enhanced cell adhesion, likely through interaction with nucleolin.

## 4. Discussion

*A. phagocytophilum* infection enhances the adhesion of neutrophils to infected endothelial cells [4,9]. In this study, it was found that AFAP enhanced cell adhesion in transfected cells. Cell adhesion is mediated by adhesion molecules located on the cell surface. AFAP was found localized to the cell periphery at 48 and 72 h post-transfection, indicating that it may participate in cell adhesion. Cell adhesion between the cell and the extracellular matrix is mainly mediated by junctional contact, such as focal adhesion and podosomes [15,16]. Podosomes are characterized with two-part architecture, in which F-actin-rich cores are surrounded by rings composed of adhesion plaque proteins and integrins [16,28]. In AFAP-expressing cells, the structures presented as F-actin-rich cores surrounded by AFAP rings were observed. Whether these structures had the characteristics of podosomes and whether they were formed in infected cells needs further investigation.

In this study, host nucleolin was identified as an AFAP-interacting protein. The nucleolin–AFAP interaction location was likely on the cell surface, since nucleolin colocalized with AFAP in immunofluorescence labeling was stained without the permeabilization of cell membranes. Although nucleolin is mainly found in the nucleus and cytoplasm, it has also been found on the cell surface [31,32]. Cell surface nucleolin is a receptor for multiple ligands, such as laminin-1, L-selectin, and virus surface proteins, mediating cell adhesion and pathogen entry [35,36,43]. L-selectin is abundantly expressed on circulating neutrophils, and nucleolin is present on the cell surface of endothelial cells [32,44]. It is worthwhile to determine the role of nucleolin in cell adhesion between infected endothelial cells and neutrophils. Furthermore, it was reported that cell surface-expressing nucleolin is associated with actin filaments, likely through the interaction with the myosin-heavy chain 9 [45,46]. Thus, AFAP is associated with the host actin filament, likely through nucleolin and myosin-heavy chain 9 interaction. Meanwhile AFAP was found surrounding F-actin-rich structures. It is also likely that nucleolin is colocalized with the F-actin-rich structures surrounded by AFAP.

It was reported that *afap* is transcribed both in mammalian host cells and tick cells in *A. phagocytophilum* infection, and the transcription activity is much higher in tick cells than in HL-60 cells [47]. Given the multiple types of cells that are involved in the migration of *A. phagocytophilum* in ticks, i.e., *A. phagocytophilum* migrates from midgut cells through hemocytes to the salivary glands [3], enhanced cell adhesion may facilitate the dissemination of *A. phagocytophilum* in tick cells. Furthermore *A. phagocytophilum* infection causes a dramatic change in tick actin cytoskeleton, as demonstrated by the disruption of stress fibers, which is similar to the AFAP-induced remodeling of the actin cytoskeleton in mammalian cells. It is worth investigating the role of AFAP in the disruption of stress fibers and cell adhesion in tick cells.

## 5. Conclusions

We found AFAP localized to the cell periphery, surrounded punctate F-actin-rich cores and enhanced cell adhesion. Host nucleolin was identified as an interacting protein of AFAP. Disruption of nucleolin attenuated AFAP-mediated cell adhesion, indicating that AFAP enhanced cell adhesion, likely through interaction with nucleolin. This study may help understand the mechanism underlying *A. phagocytophilum*-promoting cell adhesion, facilitating the elucidation of HGA pathogenesis.

## Data Availability

Not applicable.

## Supplementary Materials

The following supporting information can be downloaded at: https://www.mdpi.com/article/10.3390/jpm13020302/s1. Figure S1: Pull-down assay; Figure S2: Cell detachment assay.
