# Supplementary material for: Enhancement of Cell Adhesion by Anaplasma phagocytophilum Nucleolin-Interacting Protein AFAP
Source: J Pers Med. 2023 Feb 8;13(2):302. doi: 10.3390/jpm13020302 (PMC9965380; doi:10.3390/jpm13020302)

Supplementary Materials and Methods

1. Pull-down assay

HEK293 cells stably expressing AFAP-SF, SF tags, or APH0215-SF were cultured in one T25 culture flask. ~ 7 ×10^6 cells were harvested by centrifugation at 250× *g* for 5 min and lysed in 1 mL lysis buffer (30 mM Tris-HCl, 150 mM NaCl, and 0.5% (*v/v*) nonidet-P40, pH 7.4), supplemented with protease inhibitor cocktail and phosphatase inhibitors (APExBIO, Houston, TX, USA). The cell lysates were cleared by centrifugation at 12,000× *g* for 10 min at 4 °C, and the supernatants were subjected to incubation with 20 μL magnetic beads conjugated with mouse anti-FLAG antibody (Bimake, Shanghai, China). After incubation for 2 h at 4 °C, the beads were washed with washing buffer (30 mM Tris-HCl, 150 mM NaCl, and 0.1% (*v/v*) nonidet-P40, pH 7.4, supplemented with protease inhibitor cocktail and phosphatase inhibitors) once and TBS (30 mM Tris-HCl, 150 mM NaCl, pH 7.4) twice, followed by elution with 40 μL non-reducing 2 × SDS-PAGE sample loading buffer (100 mM Tris-Cl, 20% (*v/v*) glycerol, 4% (*w/v*) SDS, and 0.2% (*w/v*) bromophenol blue, pH 6.8). The eluates were boiled for 5 min after supplemented with 100 mM DTT and subjected to Western blot analysis. The nitrocellulose membrane with transferred proteins and prestained protein marker (ThermoFisher Scientific, Waltham, MA, USA) was cut to two pieces along the line marked with 70 kDa. One piece of membrane with larger size-proteins was probed with mouse monoclonal anti-nucleolin (D-6, 1:1000 dilution, Santa Cruz Biotechnology, Dallas, TX, USA) , the other piece of membrane with smaller size-proteins was probed with rabbit polyclonal anti-FLAG antibody (1:1000 dilution, Bioworld Technology, Bloomington, MN, USA). The incubation with primary antibodies were at RT for 1 h. After washing three times with 1 × PBS (10 min each time), the membranes were incubated with secondary antibody, peroxidase-conjugated goat anti-mouse IgG (1:2000 dilution), or peroxidase-conjugated goat anti-rabbit IgG (1:2000 dilution) (KPL, Gaithersburg, MD, USA), respectively at RT for 1 h. The membranes were washed four times with 1 × PBS (10 min each time), and subjected to ECL chemiluminescence. The membranes were imaged by Tanon 4200 chemiluminescence imaging system (Tanon, Shanghai, China).

Figure S1

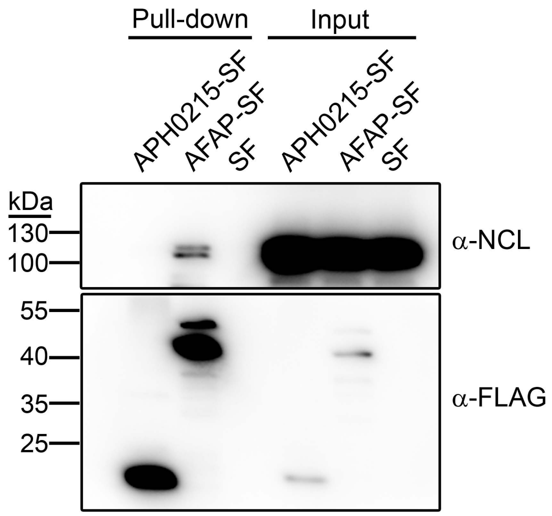

Figure S1. Pull-down assay.

Cell lysates from HEK293 cells stably expressing AFAP-SF, SF tags, or APH0215-SF, were pulled-down with anti-FLAG tag magnetic beads. Cell lysates (Input) and pulled-down products were subjected to Western blot analysis using mouse monoclonal anti-nucleolin (α-NCL) and rabbit polyclonal anti-FLAG antibody (α-FLAG). Of note, the expected size of APH0215-SF and AFAP-SF is 24.0 kDa and 41.1 kDa.

Figure S2

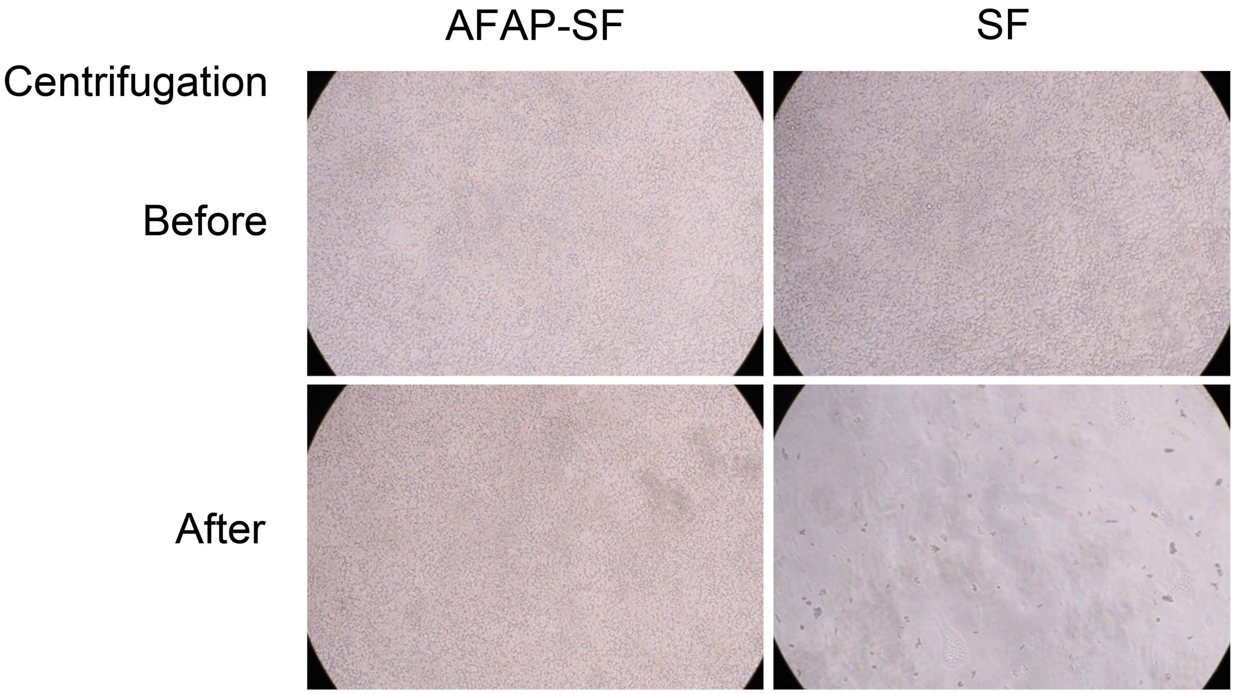

Figure S2. Cell detachment assay.
HEK293 cells stably transfected with pTAP-AFAP (AFAP-SF) or pTAP (SF) were subjected to cell detachment assay. Cell images were captured under light microscope (x100 magnification), before and after centrifugation.

Original Image for Figure 2C

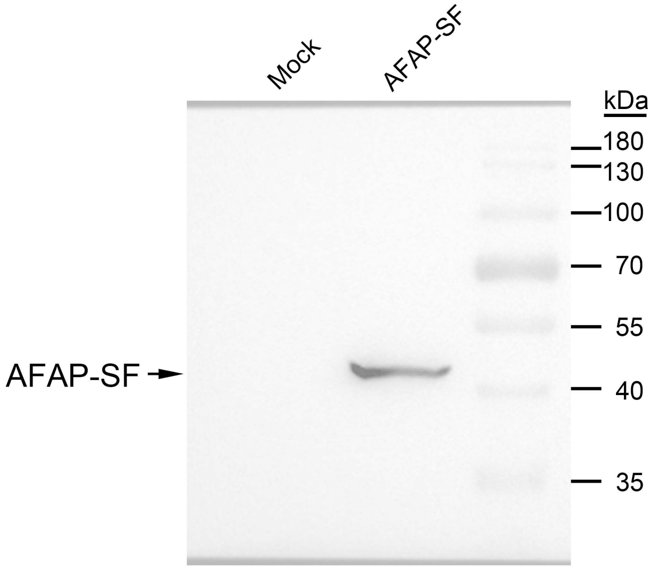

Original Images for Figure 2E-F

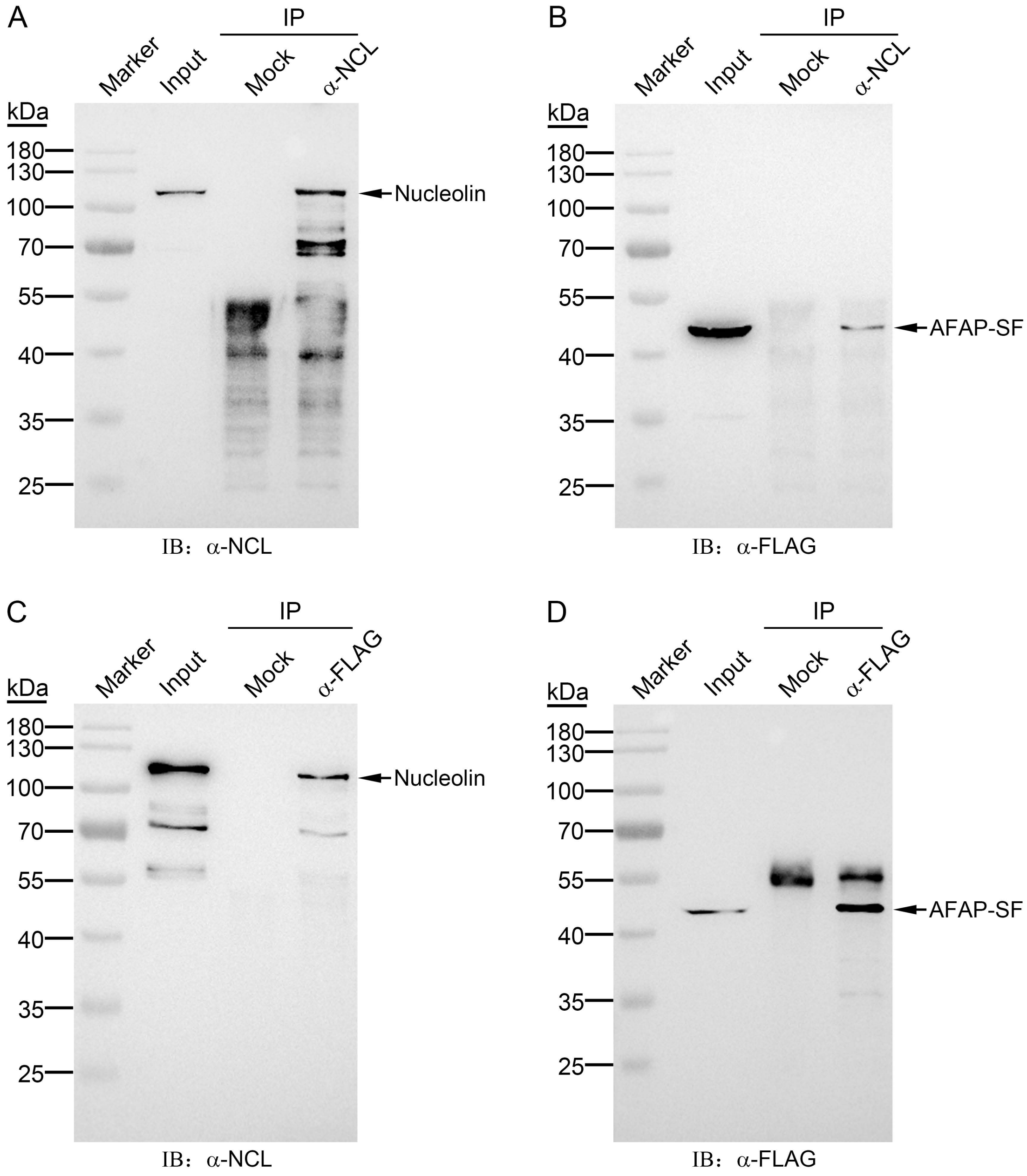

Original Images for Figure 3C

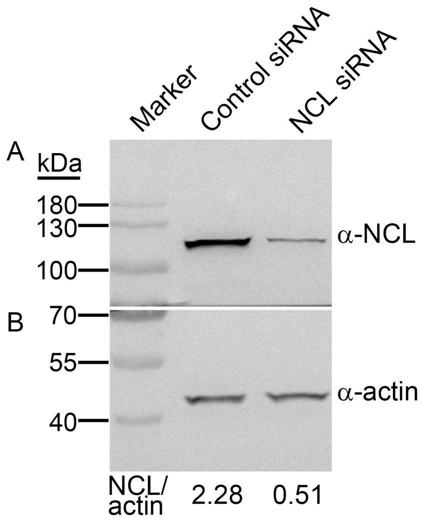

Original image for Figure S1

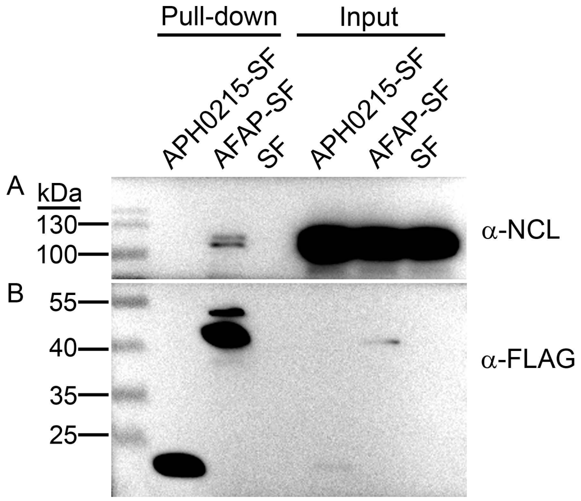

Supplement: Supplementary file 1 [file jpm-13-00302-s001.zip › jpm-2091887-supplementary.pdf]
